# Non‐coding RNAs and colitis‐associated cancer: Mechanisms and clinical applications

**DOI:** 10.1002/ctm2.1253

**Published:** 2023-05-03

**Authors:** Yijie Gu, Haizhou Zhao, Lu Zheng, Chentao Zhou, Ye Han, Airong Wu, Zhenyu Jia, Tingting Xia, Qiaoming Zhi

**Affiliations:** ^1^ Department of Gastroenterology The First Affiliated Hospital of Soochow University Suzhou China; ^2^ Department of General Surgery The First Affiliated Hospital of Soochow University Suzhou China

**Keywords:** clinical applications, colitis‐associated cancer, non‐coding RNAs, regulatory mechanisms

## Abstract

**Background:**

Colitis‐associated cancer (CAC) is one of the most severe complications of inflammatory bowel disease (IBD), which has caused a worse survival rate in IBD patients. Although the exact aetiology and pathogenesis of CAC are not completely elucidated, evidence indicates that non‐coding RNAs are closely involved and play a key role.

**Methods:**

This review aims to summarise the major findings of non‐coding RNAs in the development of CAC and present the potential mechanistic links between non‐coding RNAs and CAC pathogenesis. The results show that non‐coding RNAs can hinder DNA mismatch repair proteins and obstruct chromosome passenger complexes to increase microsatellite instability and accumulate chromosomal instability, respectively. The data also suggest that DNA promoter methylation or RNA methylation modifications of non‐coding RNA are the main mechanisms to regulate oncogene or tumour suppressor expression during the CAC progression. Other factors, including gut microbiota perturbations, immune dysregulation and barrier dysfunction, are also regulated and influenced by non‐coding RNAs. Besides, non‐coding RNAs as molecular managers are associated with multiple critical signalling pathways governing the initiation, progression and metastasis of CAC, including the janus kinase/signal transducer and activator of transcription (JAK/STAT), nuclear factor‐kappa B (NF‐κB), extracellular signal‐regulated kinase (ERK), Toll‐like receptor 4 (TLR4), Wnt/β‐catenin and phosphatidylinositol 3‐Kinase/Protein Kinase B (PI3K/AKT) pathways. In addition, non‐coding RNAs can be detected in colon tissues or blood, and their aberrant expressions and diagnostic and prognostic roles are also discussed and confirmed in CAC patients.

**Conclusions:**

It is believed that a deepening understanding of non‐coding RNAs in CAC pathogenesis may prevent the progression to carcinogenesis, and will offer new effective therapies for CAC patients.

## Graphical abstract

Highlights

1. Non‐coding RNAs are closely involved and play a key role in the pathogenesis of colitis‐associated cancer (CAC) through different mechanisms.

2. Non‐coding RNAs have been investigated singly or in panels to evaluate their diagnostic and prognostic potentials in CAC patients.

3. Emerging technologies and newly developed approaches will help us identify novel non‐coding RNAs and distinguish patients with CAC.

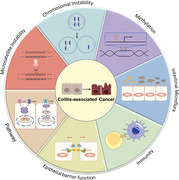

## 1 INTRODUCTION

Colorectal cancer (CRC) has become the third most common cancer and ranks second in terms of mortality in the world, causing approximately 570 000 deaths every year. The global incidence has risen to more than 1.9 million new individuals by 2020.^1^ Sporadic colorectal cancer (sCRC) is a frequent form of CRC, which accounts for >90% of CRC cases, and usually results from continuous accumulation of genomic alterations. Compared to the more common polyp‐induced sCRC, colitis‐associated cancer (CAC) is a rare type of CRC, with specific characteristics that tend to impose a less favourable prognosis.^2^, ^3^ It is one of the most important contributors of morbidity and mortality among patients with inflammatory bowel diseases (IBD), such as Crohn's disease (CD) and ulcerative colitis (UC).^4^, ^5^ The risk of CRC in IBD patients is roughly two to five times than that of the general population.^6^, ^7^, ^8^ For example, a landmark meta‐analysis from Eaden et al. reported that the cumulative probabilities of CRC in any patients with UC are 2% by 10 years, 8% by 20 years and 18% by 30 years.^9^ Moreover, mounting evidence demonstrates that cancer in IBD patients has a higher mortality rate and appears at a younger age, compared to sCRC.^10^, ^11^, ^12^

**FIGURE 1:**
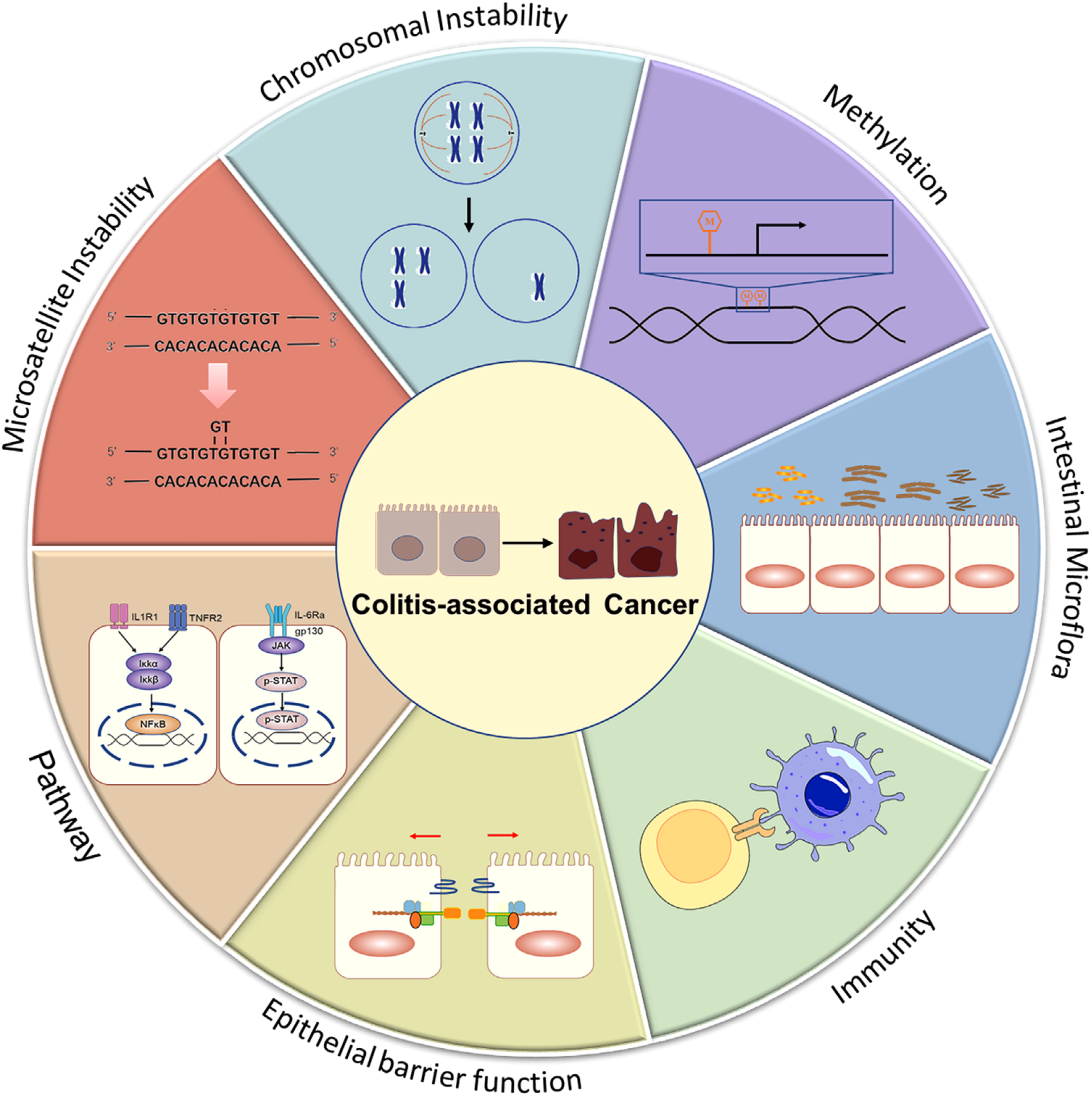
The exact aetiology and pathogenesis of colitis‐associated cancer (CAC) are closely associated with multiple factors, including microsatellite instability (MSI), chromosomal instability (CIN), DNA/RNA methylation, gut microbiota perturbations, intestinal immune dysregulation, barrier dysfunction and activation of multiple critical signalling pathways.

Compared with sCRC, CAC does not display a well‐established ‘mucosa–adenoma–carcinoma’ sequence in its pathogenesis. On the contrary, a distinctive ‘mucosa–dysplasia–carcinoma’ sequence can be found.^13^ Although sCRC and CAC share many similar genetic mutations, there are still some differences in critical genes and pathways that can distinguish these two CRC types. For instance, in sCRC patients, early mutations of the adenomatous polyposis coli (APC) tumour suppressor gene can be determined to activate the Wnt/β‐catenin pathway, whereas APC gene mutations are late events during the CAC progression. β‐Catenin mutations are rarer in CAC patients, and mutations in p53 and K‐ras occur rather earlier during the disease progression.^14^ From a molecular perspective, genetic and epigenetic abnormalities, such as microsatellite instability (MSI), chromosomal instability (CIN) and DNA/RNA methylation, are thought to be responsible for IBD‐associated dysplasia progression to cancer.^13^, ^15^, ^16^ Recent data also indicate that dysfunctions of cellular barriers or immunity are closely involved, and intestinal microbiota also seem to play an essential role in the pathogenesis of CAC.^17^, ^18^ Besides, several inflammatory‐related pathways, such as the nuclear factor‐kappa B (NF‐κB), janus kinase/signal transducer and activator of transcription (JAK–STAT) and Hedgehog (Hh) pathways, have been proven to be associated with the CAC pathophysiology^19^, ^20^, ^21^ (Figure 1).

Non‐coding RNAs are a common set of RNAs that lack any protein‐coding potentials. In the past decades, these non‐coding RNAs without protein‐coding capacity were mistakenly considered as ‘evolutionary junk’. However, emerging data have demonstrated that a portion of non‐coding RNAs are functional RNA molecules. These potential functional non‐coding RNAs can be classified into two categories: housekeeping non‐coding RNAs, including ribosomal RNAs (rRNAs) and transfer RNAs (tRNAs), as well as regulatory non‐coding RNAs, such as long non‐coding RNAs (lncRNAs), circular RNAs (circRNAs), microRNAs (miRNAs), PIWI‐interacting RNAs (piRNAs), tRNA‐derived small RNAs (tRFs) and small nucleolar RNAs.^22^, ^23^ As novel regulators of gene expression, regulatory non‐coding RNAs have a profound impact on cancer development and progression in many different cellular pathways and manners.^24^ For example, miRNAs, which have highly conserved and endogenous small single‐stranded segments (20–24 nucleotides), are able to post‐transcriptionally repress protein translation by inducing the messenger RNA (mRNA) degradation and/or inhibiting protein synthesis by base‐pairing to the 3′‐untranslated region (3′‐UTR) of cytoplasmic mRNAs.^25^ Except for the minimum size limit of 200 nucleotides and a lack of protein‐coding potential, lncRNAs are found to interact with DNAs, mRNAs, miRNAs and proteins, which consequently mediate gene expressions at transcriptional, post‐transcriptional or epigenetic levels.^26^ circRNAs, as a kind of novel identified non‐coding RNAs, comprise covalently closed loops without 5′−3′ polarity or polyadenine tails. Based on the higher sequence conservation than other types of RNA, circRNAs have been identified as novel and stable molecular markers in numerous cancer types. They have been shown to impact gene regulation by serving as competitors or decoys for miRNAs and proteins.^27^, ^28^ More interestingly, some of these lncRNAs or circRNAs have also been identified to encode functional small oligopeptides, which may participate considerably in the cancer progression.^29^, ^30^

IBD, including CD and UC, is considered as a class of systemic chronic inflammatory conditions that may harbour a multitude of intestinal and extraintestinal complications. For the past few decades, a close connection between IBD and non‐coding RNAs has been reported. Numerous non‐coding RNAs, including miRNAs, lncRNAs and circRNAs, have been confirmed to participate in the complex regulatory system of IBD.^31^, ^32^ Moreover, scientists have also emphasised how non‐coding RNAs manifest the IBD‐related complications.^33^ For instance, intestinal fibrosis is considered as one of the most common and severe complications for CD patients. In 2021, Li et al. reported that miR‐155 could directly target HBP1 and activate the Wnt/β‐catenin signalling pathway, which finally promoted the colitis‐associated intestinal fibrosis.^34^ Currently, the importance of non‐coding RNAs, including the emerging lncRNAs and circRNAs in intestinal fibrogenesis, is also systematically discussed. These non‐coding RNAs are dysregulated and mainly associated with the transforming growth factor‐β modulation and extracellular matrix remodelling.^35^

Perhaps another most well‐studied and non‐negligible complication of IBD is CAC, arguably a feared and potentially life‐threatening condition. During the occurrence of CAC, non‐coding RNAs have also been proven to play a significant role from non‐neoplastic mucosa to dysplastic and invasive carcinoma. The aim of our review is to discuss the major findings of non‐coding RNAs in the CAC progression and present the potential mechanistic links between non‐coding RNAs and CAC. Meanwhile, many non‐coding RNAs have already been successfully reported to act as molecular biomarkers or targeted therapies for CAC; hence, these potential molecules have also opened up a novel field of diagnostic and therapeutic opportunities.

## 2 THE REGULATORY MECHANISMS OF NON‐CODING RNAS IN CAC

### 2.1 Non‐coding RNAs suppress mismatch repair proteins to induce MSI in CAC

MSI refers to the genetic instability featured by length alternations within simple repeated microsatellite sequences. MSI is recognised as the tumourigenic pathway in approximately 10%−15% of sCRC patients and represents a molecular hallmark of hereditary CRC, such as Lynch syndrome.^36^, ^37^ The DNA mismatch repair (MMR) proteins function as DNA damage recognition and repair to decrease MSI. Human MutL homologue 1 (MLH1) and human MutS homologue 2 (MSH2) are the dominant MMR proteins. MSH2 combines with MSH6 to form heterodimers MutS, while MLH1 combines with PSM2 to form heterodimers MutL.^38^, ^39^ MMR system is recognised as an excision–resynthesis system that can be divided into four phases: (1) recognition of mismatched DNA, recruitment of repair enzymes, excision of the mismatch and DNA resynthesis. (2) MutS complex forms a sliding clamp binding double‐stranded DNA and changes its binding properties with the change in adenosine triphosphate (ATP) and adenosine diphosphate (ADP). The complex searches for mismatch in the form of a sliding clap in the presence of ADP while binding tightly to mismatches in the presence of ATP. (3) The DNA–MutS–ATP complex recruits the MutL complex, which functions as molecular matchmaker to recruit exonuclease I (Exo1), DNA polymerase, proliferating cell nuclear antigen (PCNA) and other proteins for excision. (4) Exo1 can mediate excision of mismatch and DNA polymerase repair synthesis under the enhanced effect of PCNA.^40^, ^41^

**FIGURE 2:**
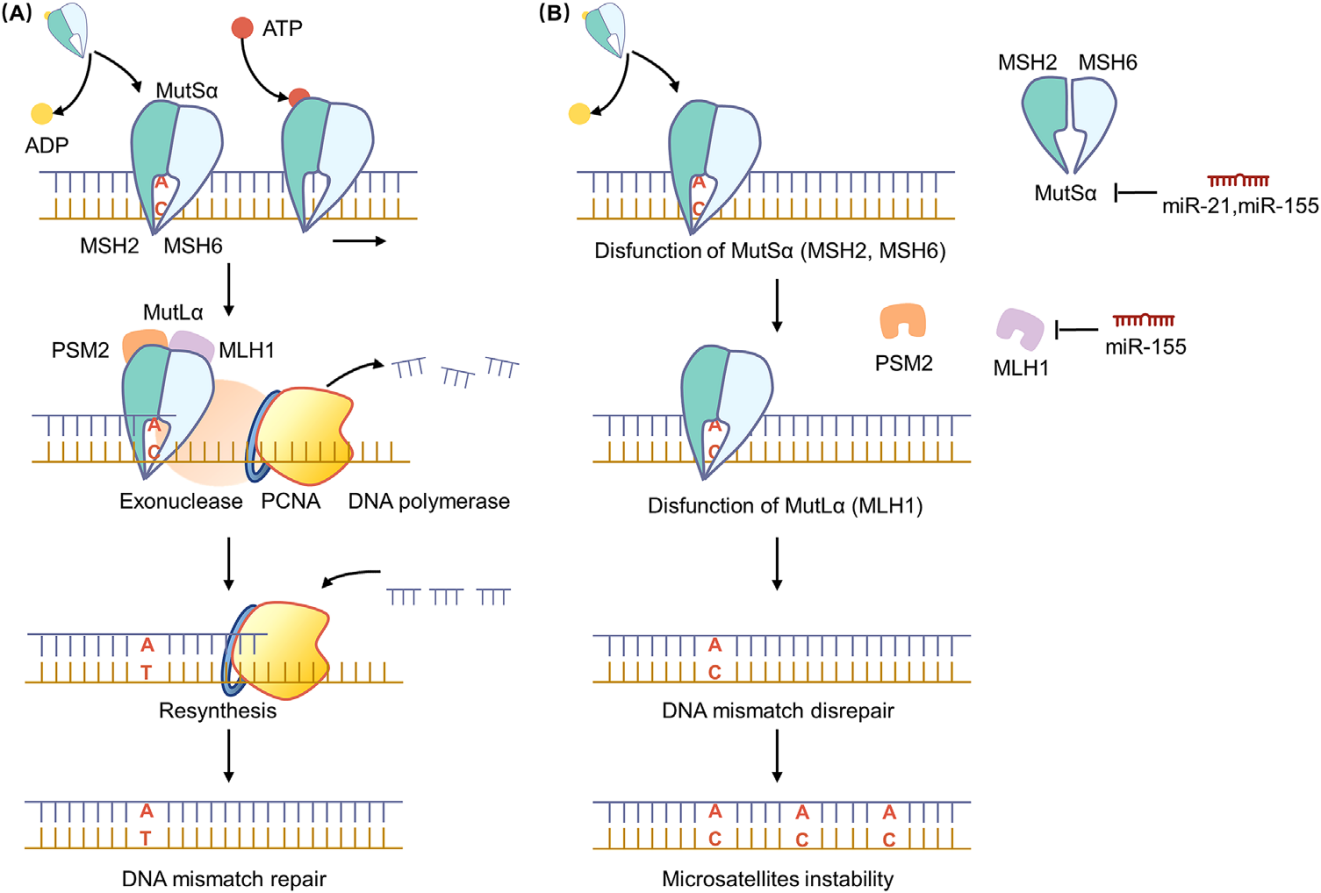
Non‐coding RNAs suppress mismatch repair (MMR) proteins to induce microsatellite instability (MSI) in colitis‐associated cancer (CAC). (A) DNA MMR proteins function as DNA damage recognition and repair to decrease MSI. (B) Oncogenic miR‐155 and/or miR‐21 can target the core MMR proteins (hMSH2, hMSH6 or hMLH1), and induce a mutator phenotype of MSI.

Previous studies have reported a wide range of MSI high in IBD‐related CRC,^42^ and the DNA promoter hypermethylation at CpG islands has been established as a common mechanism of MMR gene inactivation in IBD carcinogenesis.^37^, ^43^, ^44^ However, recent evidence has indicated that oncogenic non‐coding RNAs may also be proposed to play a significant role in modulating MMR system by down‐regulating the core MMR proteins in the CAC progression. For example, in 2013, Svrcek et al. reported that miR‐155 and/or miR‐21 over‐expression could be a pre‐neoplastic event favouring the emergence of MMR‐deficient clones in the colonic mucosa of IBD patients and thereby the occurrence of colon cancer tissues displaying MSI.^45^ In CRC cells, miR‐21 was proven to directly target the core MMR proteins (hMSH2 and hMSH6),^46^ while miR‐155 over‐expression also significantly down‐regulated the core MMR proteins (hMLH1, hMSH2 and hMSH6) inducing a mutator phenotype (MSI).^47^ Accumulating evidence has suggested that primary sclerosing cholangitis (PSC) is frequently co‐occurred by IBD, predominantly in the form of UC, and can be served as a key and additional risk factor for the colonic neoplasia in UC patients (PSC‐UC). A recent study from Adamowicz et al. showed that miR‐155 could act as a connection between inflammation and neoplasia in colitis associated with PSC, and miR‐155‐mediated suppression of MMR proteins might contribute to the colorectal neoplastic transformation in PSC patients.^48^ In all, miR‐155 and miR‐21 mainly down‐regulate the content of MMR core proteins to increase the prevalence of MSI (Figure 2).

### 2.2 Non‐coding RNAs drive CIN to promote CAC

CIN includes abnormalities in chromosome number and structures, such as reduced number, fragment deletions and translocations.^49^ In human CAC, a high frequency of CIN reflected by aneuploidy is observed, exceeding that of sCRC.^50^, ^51^ Similarly, CIN was also confirmed in 81.25% in Azoxymethane/Dextran Sulfate Sodium (AOM/DSS)‐induced murine CACs.^52^ Several pre‐clinical studies have also indicated that CIN is closely related to a rapid acquisition of multi‐drug resistance in tumour cells.^53^, ^54^ CIN+ CRC cell lines appeared to be less sensitive to anti‐cancer agents compared to diploid cells. From the meta‐analysis of CRC patients’ outcome, CIN+ was significantly associated with a poorer clinical overall survival (OS) rate relative to diploid cancers in both early‐ and late‐stage disease, when these patients received the same cytotoxic therapy.^55^ A meta‐analysis of 63 studies including 10 126 CRC patients showed that CIN tumours responded less well to 5‐fluorouracil treatment than non‐CIN tumours and that survival rates were lower in non‐CIN patients.^56^

**FIGURE 3:**
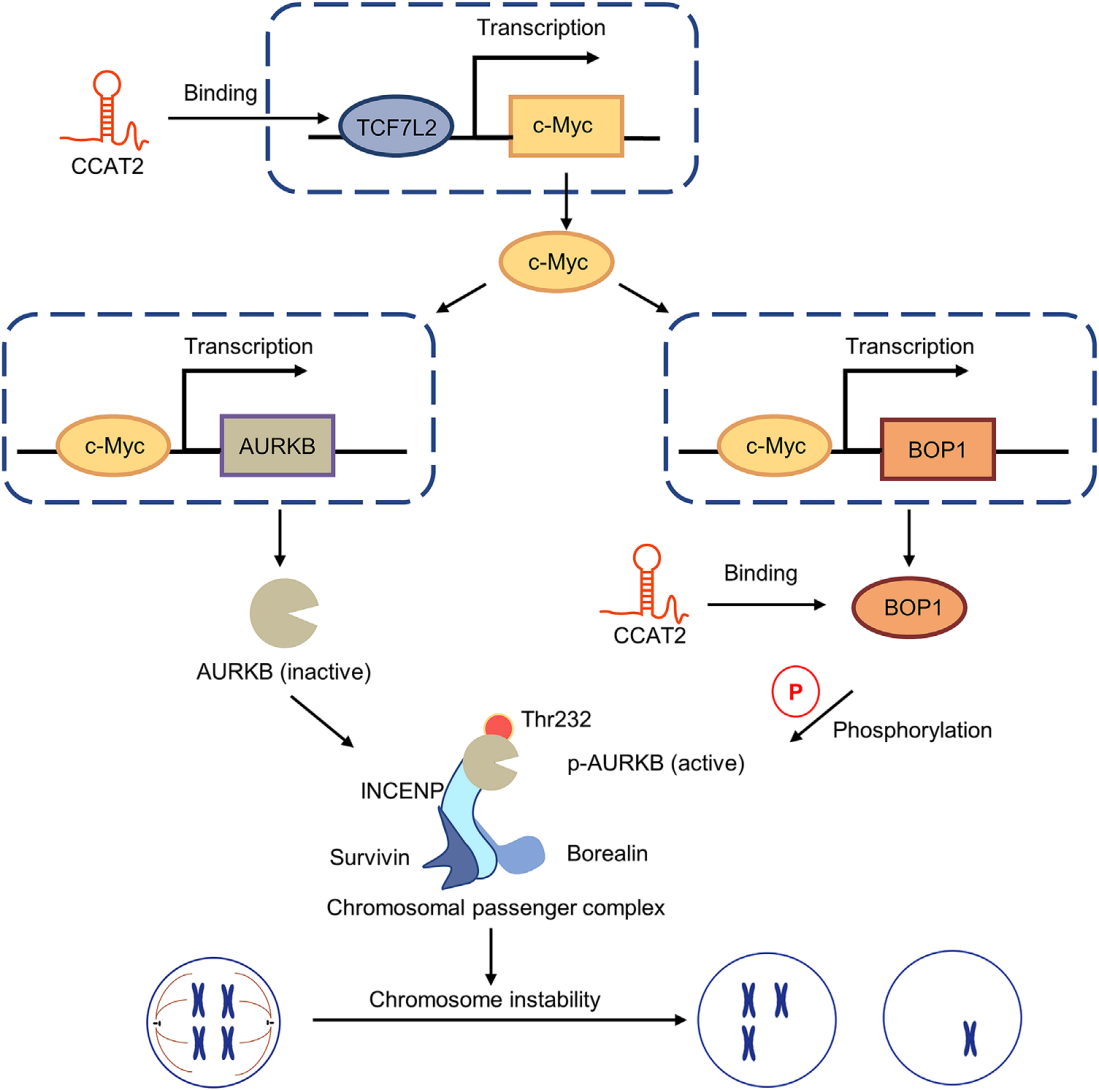
Non‐coding RNAs drive chromosomal instability (CIN) to promote colitis‐associated cancer (CAC). Long non‐coding RNA (lncRNA) CCAT2 increases the expression of MYC, which can act as a transcription factor for BOP1 and AURKB. Meanwhile, CCAT2 directly interacts with and stabilise BOP1, which can induce the phosphorylation of AURKB at Thr232, thereby reducing AURKB activity and promoting CIN.

Colon cancer‐associated transcript 2 (CCAT2) is a comprehensively investigated lncRNA that originates from the 8q24.21 chromosomal region in close proximity to the gene of MYC (300 kb upstream) and has now been studied in many malignancies as a promoter of CIN.^57^ The first indication of CCAT2 being a cancer‐related lncRNA in CRC came from the study of its encompassed SNP rs6983267.^58^ However, whether non‐coding RNAs regulate CIN in CAC remains unknown. Only one study from Chen et al. in 2020 firstly identified lncRNA–CCAT2 as a novel regulator of CIN through the BOP1 ribosomal biogenesis factor (BOP1)–Aurora kinase B (AURKB) pathway both in vitro and in vivo. They found that lncRNA–CCAT2 could effectively increase the expression of MYC, which might act as a transcription factor for BOP1 and AURKB. Meanwhile, lncRNA–CCAT2 directly interacted with and stabilised BOP1, which induced the phosphorylation of AURKB at Thr232, thereby reducing AURKB activity and promoting CIN.^59^ We propose that targeting lncRNA–CCAT2 inducing chromosomal segregation errors may provide an alternative method to suppress CIN (Figure 3). Although there is a lack of more studies nowadays, we still believe that tumour researchers will present new reliable evidence to support the roles of non‐coding RNAs in CIN in the CAC development.

### 2.3 DNA/RNA methylation mediates the non‐coding RNA expressions in CAC

**FIGURE 4:**
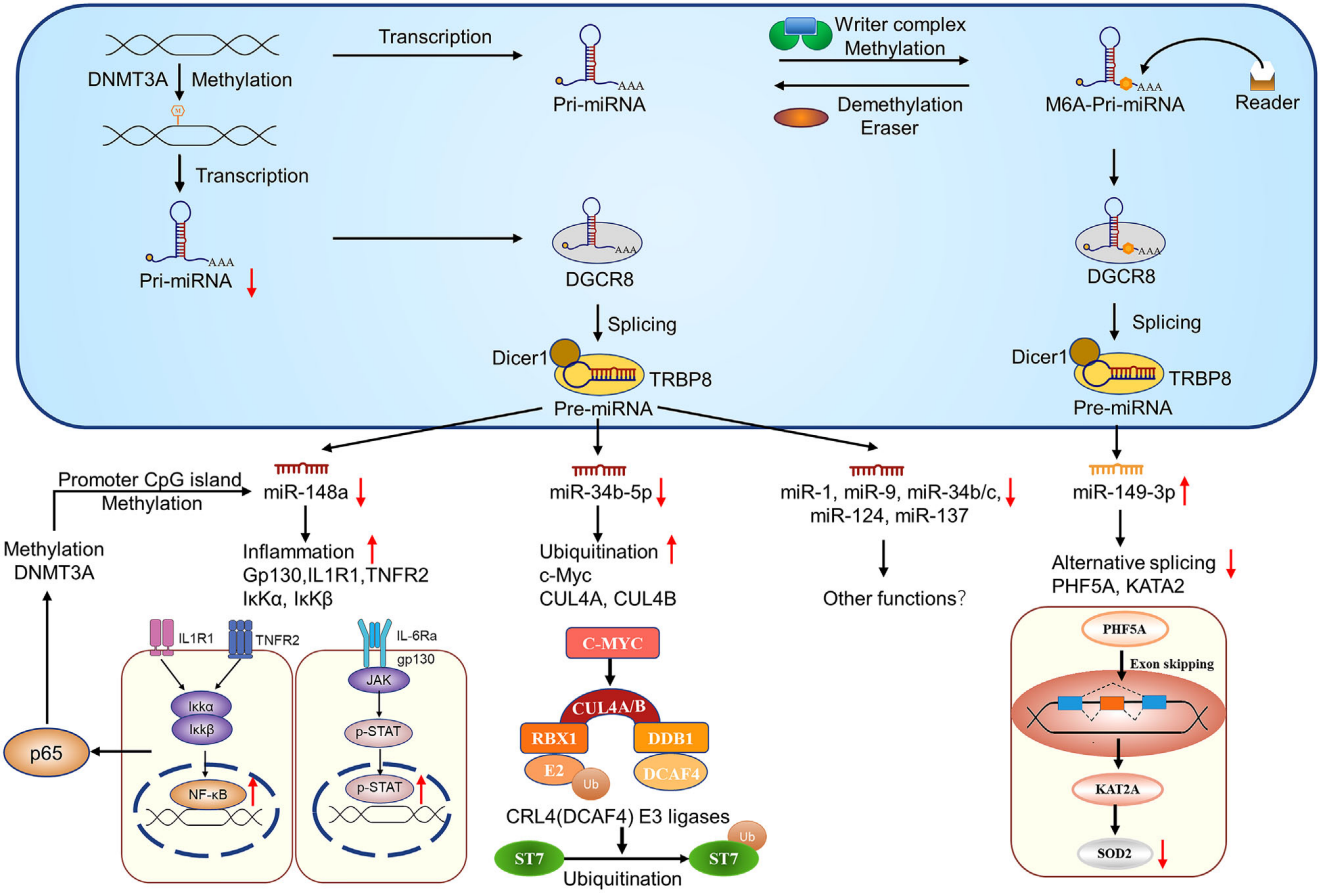
DNA/RNA methylation mediates the non‐coding RNA expressions in colitis‐associated cancer (CAC). A panel of methylated miRNAs (miR‐148a, miR‐34b‐5p, miR‐1, miR‐9, miR‐34b/c, miR‐124 and miR‐137) in their corresponding promoter regions have been proven to contribute to CAC development. Besides, the splicing process of pri‐miR‐149 is regulated by methyltransferase‐like 14 (METTL14)‐dependent m6A methylation and further promotes PHD‐finger domain protein 5A (PHF5A)‐mediated RNA alternative splicing of lysine acetyltransferase 2A (KAT2A) and transactivating superoxide dismutase‐2 (SOD2) in cells, which finally leads to the occurrence of CAC.

In the past decades, epigenetic modifications, including non‐coding RNAs, DNA methylation and chromatin modifications, have attracted more attentions as they can regulate gene expressions without altering the DNA sequence.^60^ More interestingly, emerging evidence also suggests that DNA methylation of non‐coding RNA promoters can be one of the main mechanisms by which RNA expressions are regulated.^61^ For instance, DNA methylation‐associated silencing of miR‐486‐5p could significantly promote the CRC development by activating the pleomorphic adenoma gene‐like 2/insulin‐like growth factor‐2/β‐catenin signalling pathway.^62^ lncRNA‐SLCO4A1‐AS1 was commonly up‐regulated in CRC samples and acted as an oncogenic molecule in CRC patients by mediating a Hsp90/Cdk2/c‐Myc axis. Aberrant over‐expression of SLCO4A1‐AS1 in CRC was proven to be partly due to the DNA hypomethylation of its gene promoter.^63^ In CAC, several miRNAs have also tested this hypothesis (Figure 4). In 2017, Toiyama et al. performed a validation study of clinical specimens from non‐neoplastic UC and UC‐associated dysplasia (or cancer) patients to reveal the potential methylation status of miRNAs in rectal mucosa between non‐tumour patients and patients at high risk of UC‐CRC. Eventually, a cluster of methylated miRNAs (miR‐1, miR‐9, miR‐124, miR‐34b/c, miR‐137 and miR‐148a) was proven to identify UC patients with dysplasia or cancer.^16^ In Zhu's study, miR‐148a‐deficient mice seemed to be more susceptible to colitis and CAC, and miR‐148a was also down‐regulated in human IBD and CAC patient tissues. This result might correlate with a high level of miR‐148a promoter methylation mediated by DNA methyltransferase 3 alpha (DNMT3A) and P65. Some upstream regulators, including TNFR2, IL1R1, IKKα, IKKβ and GP130, were all targets of miR‐148a, which led to the activation of NF‐κB and STAT3 pathways and increase of colitis.^64^ Another report from Yang and coworkers found that miR‐34b‐5p was significantly inhibited in cancerous colonic tissues in CAC patients, and intracellular inflammation or DNA hypermethylation could down‐regulate the miR‐34b‐5p expression, which abolished its suppressive effect on c‐MYC. The high level of c‐MYC further activated the expression of CUL4A/4B, which enhanced the ubiquitination and degradation of ST7 by CRL4DCAF4 E3 ligases, and eventually led to the CAC carcinogenesis.^65^

RNA methylation modifications, mainly including N6‐adenylation (m6A), N1‐adenylate methylation (m1A) and 5‐methylcytosine (m5C), are widely existed and involved in the biological processes of cancers.^66^, ^67^ m6A methylation modifications are removed by the demethylases (Erasers), including α‐ketoglutarate‐dependent dioxygenase homologue 5 (ALKBH5), ALKBH3, fat mass and obesity‐associated protein (FTO) and other homologies. m6A‐binding proteins called ‘Readers’ recognise these RNAs and perform their functions.^68^ Recently, Cao et al. indicated that the splicing process of pri‐miR‐149 was regulated by methyltransferase‐like 14 (METTL14)‐dependent m6A methylation. Down‐regulation of miR‐149‐3p subsequently promoted PHD‐finger domain protein 5A (PHF5A)‐mediated RNA alternative splicing of lysine acetyltransferase 2A (KAT2A) and transactivated superoxide dismutase‐2 (SOD2) in CRC cells, which finally led to the occurrence of CAC^69^ (Figure 4). These data strongly provide a potential strategy for CAC therapy by targeting RNA modification.

### 2.4 Non‐coding RNAs modulate intestinal microbiota dysbiosis in CAC

**FIGURE 5:**
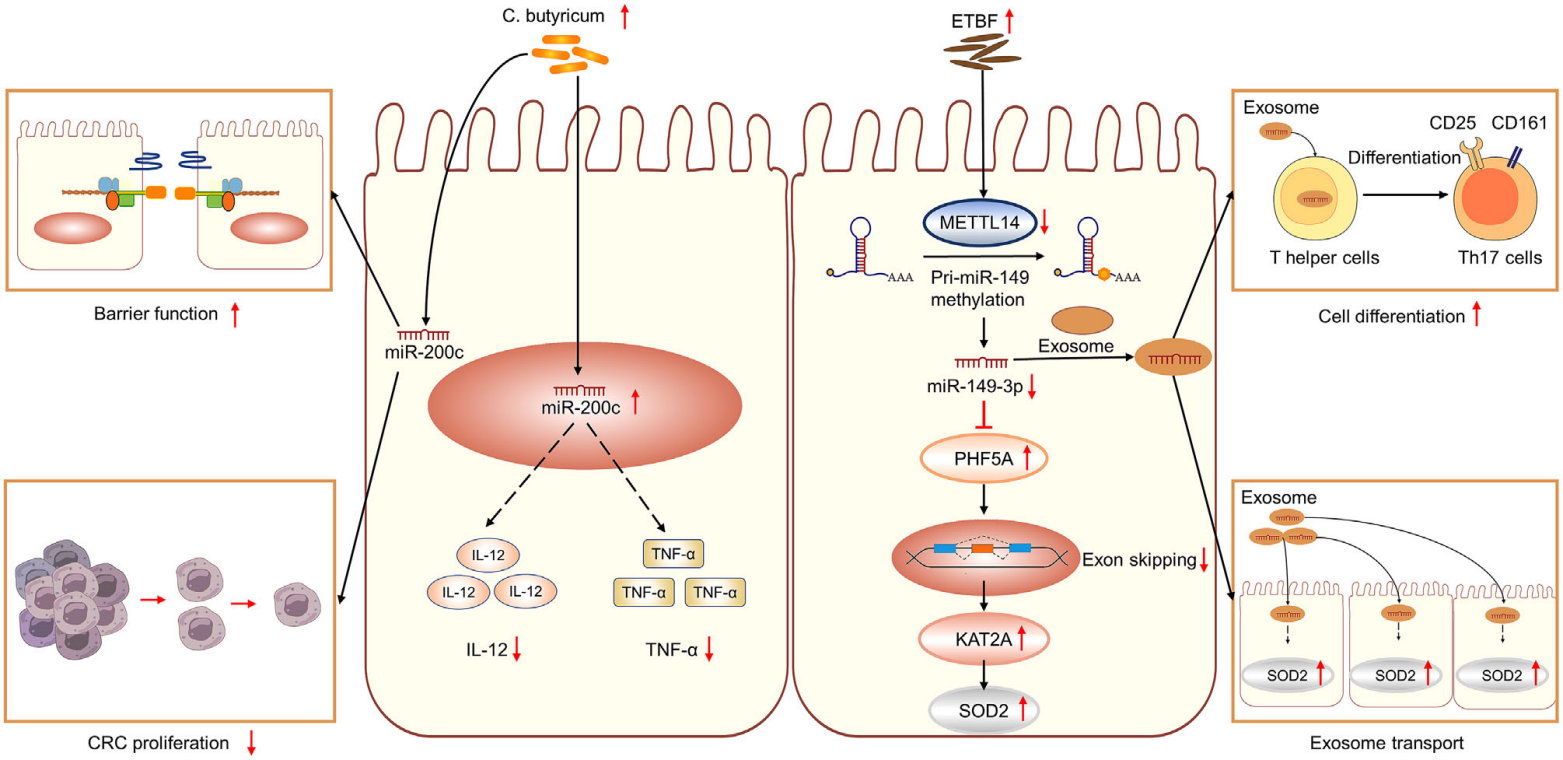
Non‐coding RNAs modulate intestinal microbiota dysbiosis in colitis‐associated cancer (CAC). *Clostridium butyricum* can attenuate the colitis‐associated cancerous responses through miR‐200c, which can suppress the colorectal cancer (CRC) cell proliferation, and decrease the intestinal permeability and reinforce various components of the colonic barrier. Up‐regulation of miR‐200c also alleviates the production of proinflammatory cytokines tumour necrosis factor‐alpha (TNF‐α) and interleukin‐12 (IL‐12). On the contrary, enterotoxigenic *Bacteroides fragilis* (ETBF)‐induced CAC carcinogenesis depended on a non‐coding RNA‐dependent mechanism. Down‐regulation of miR‐149‐3p by ETBF relies on methyltransferase‐like 14 (METTL14)‐dependent m6A methylation, which can up‐regulate a splicing modulator PHD‐finger domain protein 5A (PHF5A), and induce cell proliferation mainly through a miR‐149‐3p/PHF5A/lysine acetyltransferase 2A (KAT2A)/SOD2 axis during the CAC progression. In addition, exosomal miR‐149‐3p derived from ETBF‐treated cells can also promote the cell differentiation of T‐helper type 17.

Intestinal microbiota dysbiosis is defined as disturbances in the type, location and quantity of microorganisms such as bacteria, viruses, probiotics and fungi, resulting in a reduction of gut microbial diversity.^17^, ^70^ Dysbiosis promotes carcinogenic effects by inducing inflammation, increasing cell proliferation and producing metabolites.^70^ The gut microbiota has also been recognised and proven to potentially favour or aggravate the CAC progression. For instance, using the methods of 16S (MiSeq) and ITS2 (pyrosequencing) sequencing, an increase in enterotoxigenic *Bacteroides fragilis* (ETBF) and a decreasing level of *Fusobacterium* and *Ruminococcus* genus could be observed and characterised in CAC patients compared to sCRC patients.^71^, ^72^ Emerging data also show that the suitable manipulation of gut microbial composition using some probiotics, such as Bornlisy, *Bifidobacterium bifidum* CGMCC 15068 and *Clostridium butyricum*, can be a feasible prevention strategy for CAC.^72^, ^73^ *C. butyricum*, which is a probiotic with a variety of active products, has exerted potential positive and protective effects in humans and animals.^74^ Using the experimental model of CAC induced by AOM and trinitrobenzene sulfonic acid, Xiao et al. demonstrated that *C. butyricum* could attenuate the colitis‐associated cancerous responses through miR‐200c. *C. butyricum* could stimulate the transcription of mir‐200c, which decreased the proliferation rate of colon cancer cells. Further mechanistic studies also demonstrated that *C. butyricum* could suppress the levels of proinflammatory cytokines (tumour necrosis factor‐alpha [TNF‐α] and interleukin‐12 [IL‐12]), decrease the intestinal permeability and reinforce various components of the colonic barrier.^75^ On the contrary, ETBF is considered as a global aetiology of diarrhoeal disease in animals and humans, which is also associated with colitis.^76^, ^77^, ^78^ Furthermore, ETBF infection‐mediated inflammation is also found to significantly increase carcinogenesis in AOM/DSS‐induced wild‐type mice, which is dependent on BFT (a secreted zinc‐dependent metalloprotease toxin, 20 kDa) activity. Interestingly, Cao et al. recently performed a similar experiment, wherein ETBF‐induced CAC carcinogenesis depended on a non‐coding RNA‐dependent mechanism. The down‐regulation of miR‐149‐3p by ETBF attributed to METTL14‐dependent m6A methylation, which might up‐regulate the splicing modulator PHF5A and induce cell proliferation mainly through a miR‐149‐3p/PHF5A/KAT2A/SOD2 axis during the CAC progression. Besides, exosome‐derived and enriched miR‐149‐3p from ETBF‐treated cells could also promote the cell differentiation of T‐helper type 17 cells.^69^ These data may provide a mechanistic understanding of colitis or CAC between intestinal microbiota dysbiosis and non‐coding RNAs (Figure 5).

### 2.5 Non‐coding RNAs regulate intestinal immunity in CAC

**FIGURE 6:**
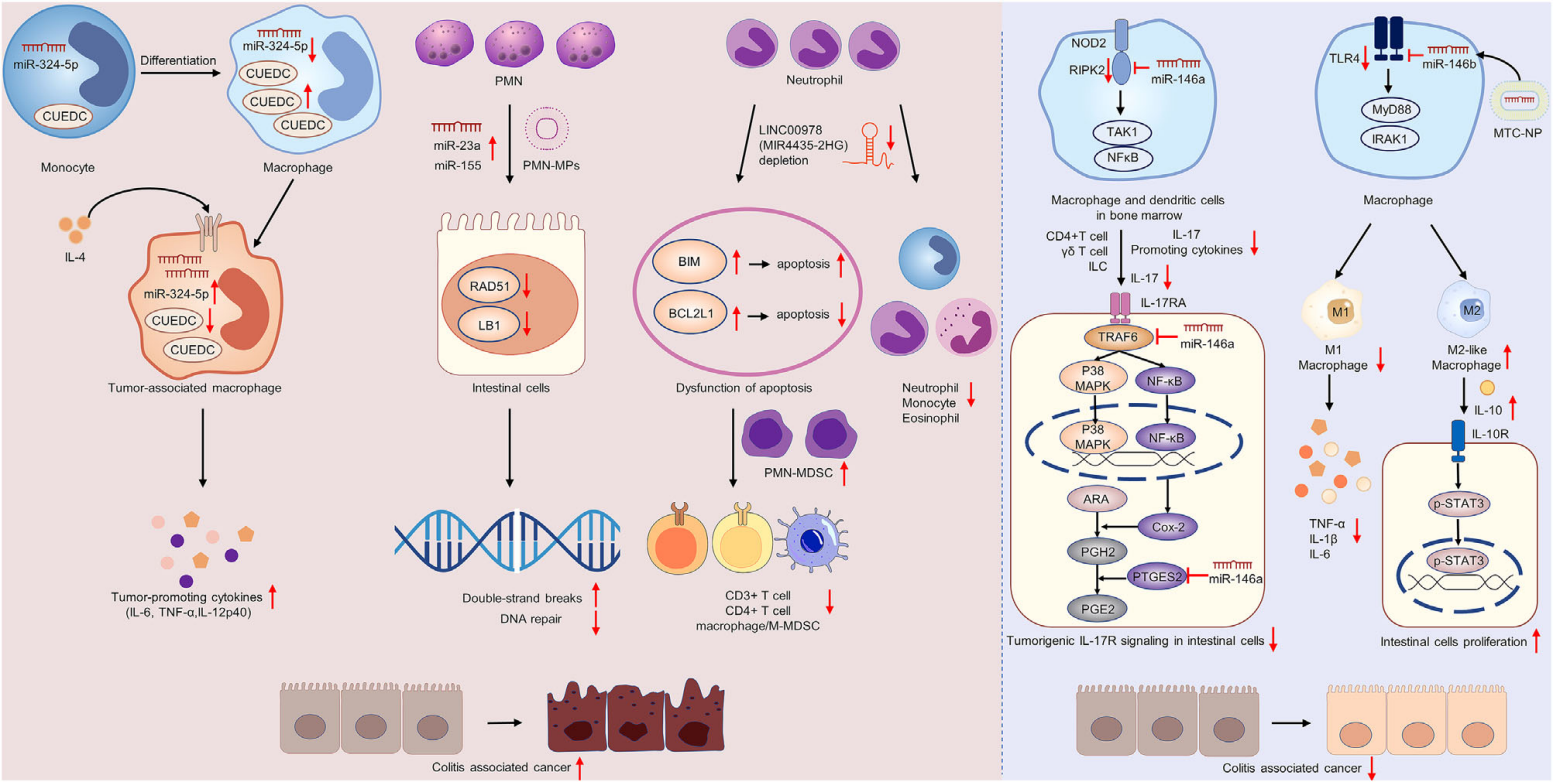
Non‐coding RNAs regulate intestinal immunity in colitis‐associated cancer (CAC). Non‐coding RNAs, including miR‐324‐5p, miR‐23a, miR‐155, LINC00978 (miR4435‐2HG), miR‐146a and miR‐146b, are crucial regulators of intestinal immunity and involved in the innate or adaptive immunity.

Concepts about CRC in IBD patients go back to the early 1900s, when Crohn et al. first reported a case of colonic adenocarcinoma in a long‐term UC patient. Nowadays, growing evidence supports the importance of innate microbial recognition by non‐immune and immune cells in the gut, and either exacerbated or diminished innate or adaptive immune responses may trigger the breakdown of intestinal homeostasis, which can finally lead to IBD or CAC.^19^, ^79^, ^80^ Tumour microenvironment coexists and interacts with various immune cells, including macrophages, neutrophils and lymphocytes, to sustain the growth of CAC cells.^81^, ^82^ In addition, T‐cell‐ or dendritic cell‐mediated immune response and release of various cytokines are also significant components in the immunopathogenesis of CAC.^80^ Moreover, recent studies have also indicated that non‐coding RNAs are crucial regulators of intestinal immunity through the innate or adaptive immunity (Figure 6). For example, miR‐324‐5p was found to regulate the differential expression of CUEDC2 during monocyte‐to‐macrophage differentiation. Elevated IL‐4 and miR‐324‐5p levels in tumour microenvironment caused a decreased expression level of CUEDC2 in tumour‐associated macrophages, which resulted in excessive levels of proinflammatory cytokines, such as TNF‐α and IL‐6, and linked with both colitis and colon carcinogenesis.^83^ Aberrant neutrophil (polymorphonuclear [PMN]) infiltration within the colonic mucosa is identified as a hallmark of IBD and is closely connected with CAC tumourigenesis.^84^, ^85^ In 2019, Bui and Sumagin discussed a novel mechanism that PMN‐derived microvesicles or microparticles (PMN‐MPs) could effectively deliver miR‐23a and miR‐155 onto the intestinal mucosa. miR‐23a and miR‐155 might down‐regulate LB1 and Rad51, respectively, which led to replication fork collapse and homologous recombination inhibition, and thereby finally drove the transformation from IBD to CAC.^85^

Conversely, LINC00978 (also known as miR4435‐2HG) was mainly located in the CAC tumour stroma and played as a tumour suppressor molecule by remodelling the immune microenvironment. LINC00978 depletion in neutrophils, but not in intestinal epithelial cells (IECs), could lead to a decline of monocytes, neutrophils and eosinophils, and increase tumour‐infiltrating PMN myeloid‐derived suppressor cells (PMN‐MDSC) to promote the cancer development.^86^ In 2021, a report from Garo et al. also demonstrated that miR‐146a was a key negative mediator in colonic inflammation and associated cancer by two interlinked mechanisms as follows: (1) in myeloid cells, miR‐146a could target RIPK2 (a NOD2 signalling intermediate) to decrease myeloid cell‐derived IL‐17‐inducing cytokines and restrict the levels of colonic IL‐17. (2) Meanwhile, in IECs, miR‐146a targeted TRAF6 to suppress IEC responsiveness to IL‐17. miR‐146a in IECs also suppressed CAC by directly inhibiting PTGES2, an essential enzyme that converted PGH2 to PGE2. Thus, IEC‐specific deletion of miR‐146a promoted the CAC progression.^87^ Similarly, miR‐146b was also regarded as a tumour suppressor candidate, and strongly suppressed M1 macrophage activation by inhibiting the Toll‐like receptor 4 (TLR4) signalling pathway and proinflammatory cytokine induction. Meanwhile, miR‐146b mediated the macrophages to differentiate into M2‐like phenotype cells to promote the growth of epithelial cells by the STAT3‐dependent IL‐10 production and hampered the development of CAC after DSS‐induced injury.^88^

As described above, we provide an overview of different immune cells that can influence the inflamed mucosa or epithelial cells through different key non‐coding RNAs and describe how immune cells are critical to colon epithelial cells and impact the cancer development. On the other hand, as IBD‐derived dysplasia or CAC develops in the background of persistent chronic inflammation, impaired mucosa or epithelial cells can also recruit various immune cells, including monocyte‐derived macrophages, neutrophils, lymphocytes and eosinophils, to eliminate neoplastic cells.^89^ However, no evidence has been reported that these regulatory non‐coding RNAs participate in the recruitment of immune cells by epithelial cells. Thus, in particular, more studies are required to provide a detailed mechanism of how changes of non‐coding RNAs in epithelial cells impact the recruited immune cells during the CAC initiation and development.

### 2.6 Non‐coding RNAs alter intestinal epithelial barrier in CAC

IECs, which provide a selectively permeable barrier between the internal tissues and external environment, are considered as the first defensive line against infections or injuries. The mechanical junctions between neighbouring cells can effectively maintain intestinal homeostasis by permitting the absorption of nutrients and water while excluding pathogens, toxic substances or dietary antigens. Of these junctional components, the tight junctions and adhesion junctions have been highly studied and are composed of greater than 40 proteins, including claudins, occludin, cadherins and nectins.^90^ Now a defective intestinal epithelial barrier, which can increase the intestinal permeability and trigger compensatory immune responses, has been implicated as an important pathogenic factor in the initiation of colitis‐related diseases.^91^ For instance, miR‐223 was proven to be a proinflammatory molecule associated with the IL‐23 pathway. By directly targeting CLDN8, miR‐223 impaired the intestinal barrier, leading to the development of IBD.^92^

**FIGURE 7:**
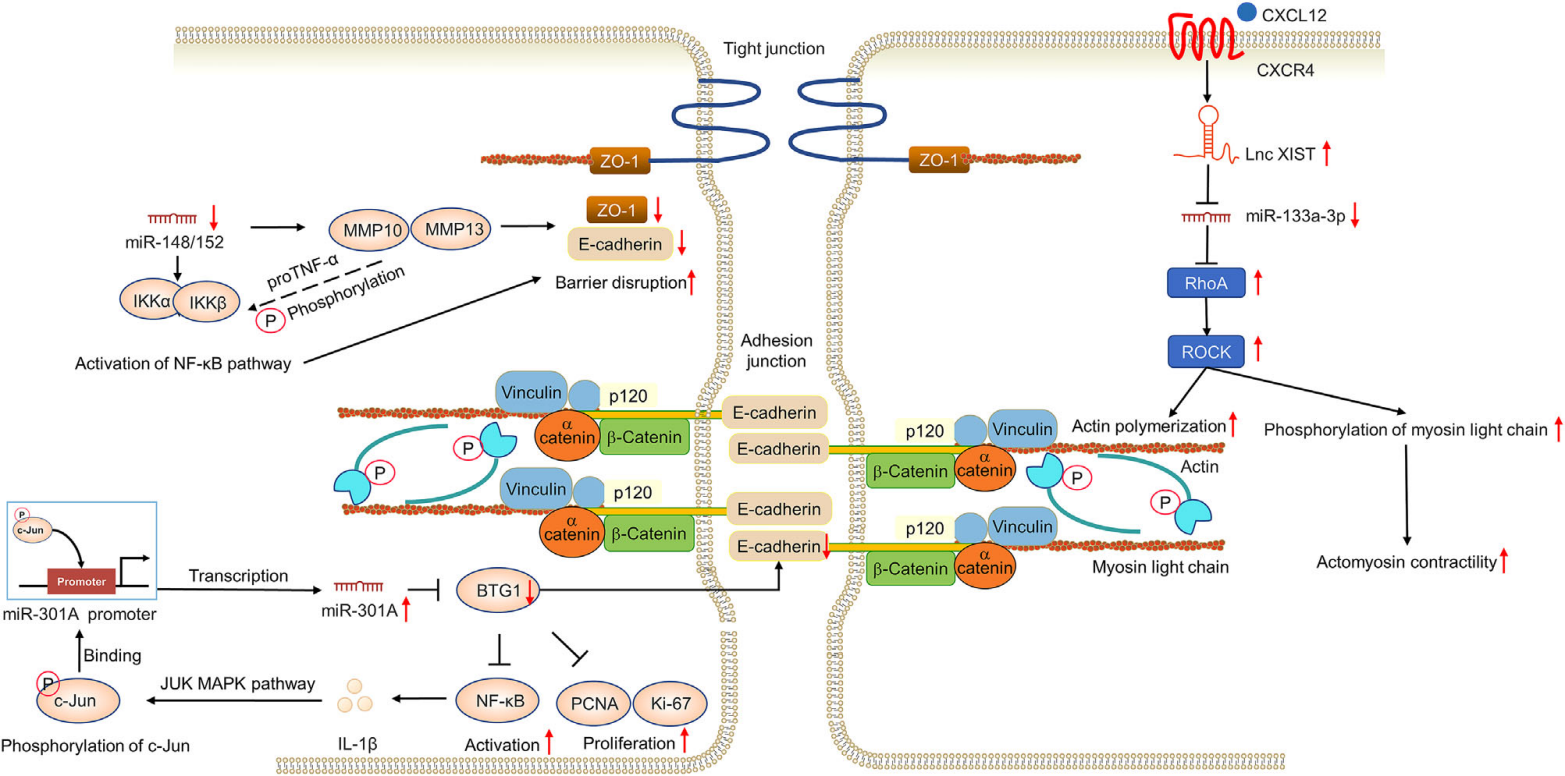
Non‐coding RNAs alter intestinal epithelial barrier in colitis‐associated cancer (CAC). Non‐coding RNAs, such as miR‐301A, miR‐148/152 and long non‐coding RNA (lncRNA) XIST, are linked to the CAC progression for actin cytoskeletal reorganisation that drives cell motility and invasion or reduced levels of the tight and adhesion junction‐related proteins.

More importantly, recent studies have implied that altered expressions of non‐coding RNAs also link with the pathogenesis of CAC via disrupting the intestinal barrier function (Figure 7). In the IEC of active IBD and CAC patients, IL‐1β was found to activate c‐jun, which was able to bind to the promoter site of miR‐301A and initiate its transcription. Mechanistically, miR‐301A could directly target and down‐regulate BTG1 to aggravate intestinal inflammation by activating NF‐κB signalling pathway and impairing barrier function. In addition, suppression of BTG1 led to the promotion of CRC cell proliferation, conferring on miR‐301A as a potential oncogene in the CAC progression.^93^ Based on knockout (KO) mice, Tang et al. demonstrated that deficiency of individual miR‐148/152 family members or full‐family aggravated the colitis or CAC, causing the disruption of intestinal barrier by mediating MMP10 and MMP13, and cleaving pro‐TNF‐α into bio‐active TNF‐α fragments to activate the NF‐κB signalling pathway. Subsequent data also revealed that the impaired barrier function in miR‐148/152 family member KO mice coupled with reduced levels of the tight junction‐ and adhesion junction‐related proteins, such as E‐cadherin, ZO‐1 and occludin.^94^ Similar to the role of miRNAs, increasing data also highlight that lncRNA is linked to the CAC progression for actin cytoskeletal reorganisation that drives cell motility and invasion. Functionally, CXCL12/CXCR4‐induced up‐regulation of lncRNA XIST might function as a ceRNA to effectively sponge miR‐133a‐3p, thereby driving the cancer progression through a RhoA/ROCK/p‐MLC pathway.^95^

### 2.7 Non‐coding RNAs mediate multiple signalling pathways governing CAC

It is well established that chronic and long‐standing intestinal inflammation is a significant essential factor of CAC. Compared to sCRC, IBD‐associated CRC (CAC) may emerge through distinct pathways of carcinogenesis.^20^ Of these various signalling pathways involved in colonic inflammation and CAC, the NF‐κB and JAK/STAT pathways are central.^96^, ^97^, ^98^ Notably, the STAT3 and NF‐κB signals are found to be hyperactive during the whole process, while other signals, such as the p38 MAPK and Wnt/β‐catenin signals, are only hyperactive at the beginning and end of stage, respectively.^99^ However, different conclusions are present. For instance, in 2014, Li et al. used microarray to examine the mRNA expression profiles among different groups of mice and demonstrated that the Wnt signalling was persistently activated in the whole process of CAC by Kyoto Encyclopedia of Genes and Genomes analyses. But their findings lacked of enough follow‐up experimental data in vitro and in vivo.^100^ Unlike their conclusions, our recent study screened the changes in potential signalling pathways during the whole process from normal colon mucus, colitis to CAC through RNA sequence, and demonstrated that the Wnt/β‐catenin signalling was only hyperactive during the colitis‐to‐carcinoma transition, which implied that the Wnt signals might bridge the potential gap between chronic intestinal inflammation and carcinogenesis.^101^ Despite the controversies, these significant signalling pathways can be widely activated in IBD patients, and collaboratively contribute to the CAC carcinogenesis by stimulating cell proliferation, angiogenesis and invasion.

**FIGURE 8:**
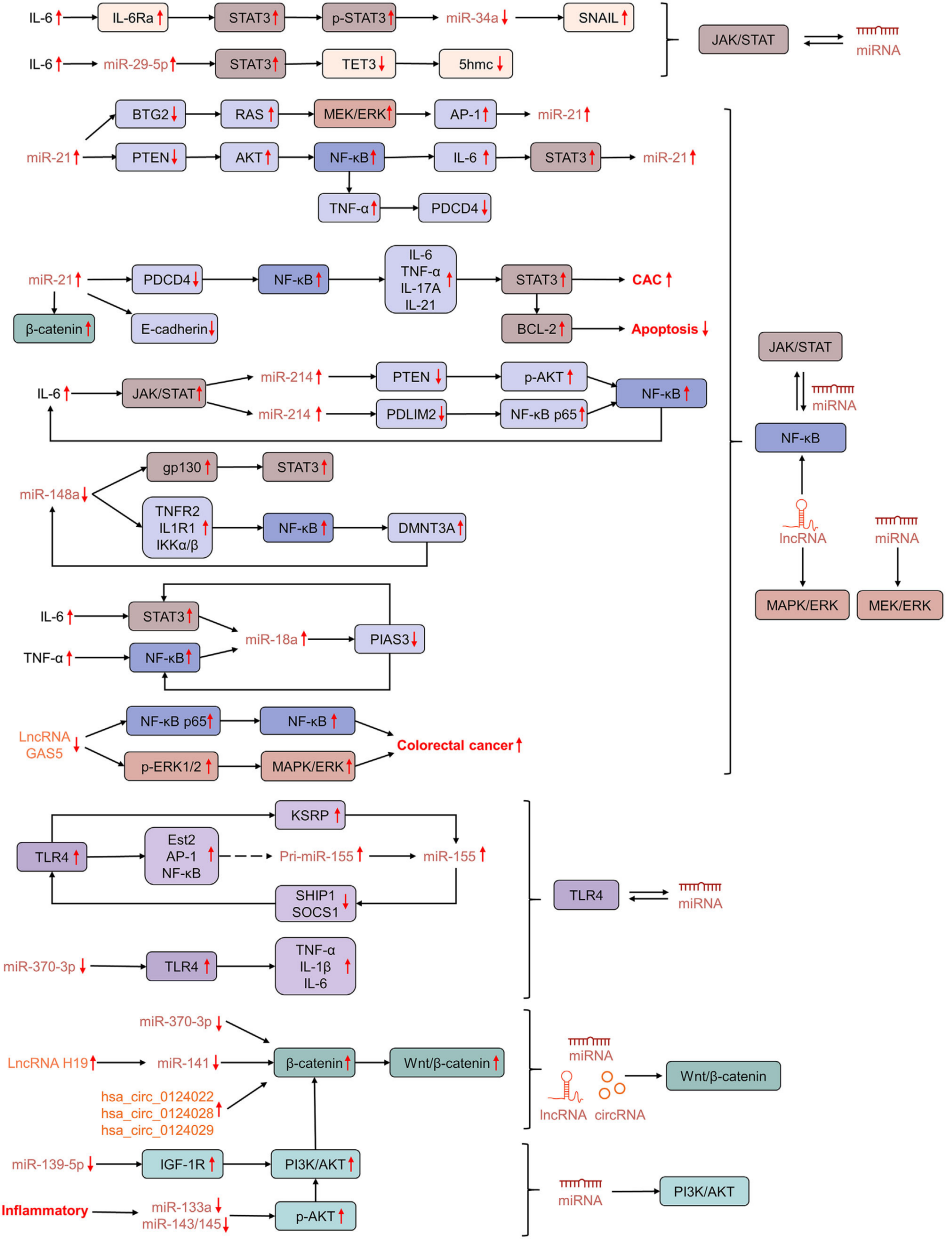
Non‐coding RNAs mediate multiple signalling pathways governing colitis‐associated cancer (CAC). Of these various signalling pathways, the janus kinase/signal transducer and activator of transcription (JAK/STAT), nuclear factor‐kappa B (NF‐κB), extracellular signal‐regulated kinase (ERK), Toll‐like receptor 4 (TLR4), Wnt/β‐catenin and phosphatidylinositol 3‐Kinase/Protein Kinase B (PI3K/AKT) pathways are central and can be mediated by non‐coding RNAs.

Numerous studies have documented that these colon inflammation or CAC‐related signals are also regulated or activated by some specific non‐coding RNAs (miRNAs) (Figure 8). Taking the JAK/STAT pathway as an example, the cytokine IL‐6 was proven to activate the oncogenic STAT3 transcription factor, which could repress the MIR34A gene directly through the phylogenetically conserved STAT3‐binding site in the first intron. An activated IL‐6R/STAT3/miR‐34a feedback loop was required and essential for maintaining the mesenchymal phenotype of CRC and colitis‐associated intestinal tumours.^102^ Similarly, in the development of CAC, stimulating inflammatory factor IL‐6 might also induce miR‐29a up‐regulation in both epithelial cells and immune cells, which could increase STAT3 expression and lead to the TET3 and 5hmC decrease in epithelial cells.^103^ Evidence also indicates that members of the JAK/STAT, NF‐κB or other signalling pathways are suspected to crosstalk, and the complex networks of signals can be a crucial link between persistent chronic intestinal inflammation and CAC initiation. For instance, the aberrations of miRNAs can modulate STAT3 or NF‐κB activity simultaneously by targeting the members of JAK/STAT3 and NF‐κB pathways, which can promote or suppress the colitis or colitis‐associated tumourigenesis.^64^, ^104^, ^105^, ^106^, ^107^ Meanwhile, miRNAs also interacted with some other conserved signalling pathways, such as TLR4, Wnt//β‐catenin and phosphatidylinositol 3‐Kinase/Protein Kinase B, to influence cancer cell proliferation, metastasis or maintenance of cancer cell stemness.^108^, ^109^, ^110^, ^111^

In addition, collective data suggest that other types of non‐coding RNAs, such as lncRNAs and circRNAs, are also highly involved and crucial for maintaining the tumour‐initiating characteristics or potential of CAC (Figure 8). lncRNA GAS5, which is 651 nucleotides in length and located on chromosome arm 1q25, was first reported to be a negative lncRNA of the genesis and development of CAC. It is possible that down‐regulation of GAS5 can activate the NF‐κB and MAPK/extracellular signal‐regulated kinase (ERK) pathways to enhance the secretion of inflammatory cytokines in tumour cells.^112^ Recent evidence has indicated that exosomes are essentially involved in the dynamic crosstalk between primary tumour cells and microenvironment to mediate tumour initiation, progression and metastasis.^113^, ^114^ lncRNA H19 was found to be transferred from carcinoma‐associated fibroblasts (CAFs) to tumour cells through exosomes, and exosome‐derived lncRNA H19 could activate the β‐catenin pathway as a ceRNA sponge for miR‐141 in CRC and AOM/DSS‐treated CAC mouse model.^115^ Likewise, our investigation also showed that the Wnt/β‐catenin signalling was essential to the malignant transformation of colitis, and PRKAR2A‐derived circRNAs could significantly promote the colitis‐to‐CAC transition by activating the canonical Wnt signalling pathway.^101^

## 3 CLINICAL APPLICATION OF NON‐CODING RNAS IN CAC PATIENTS

As described above, CAC arises as a complex multi‐stage process starting with persistent inflammation and can be influenced by genetic or immunologic factors, and eventually displayed differences when compared with sCRC. Altered expressions of non‐coding RNAs in CAC patients were first reported by Kanaan et al. in 2012, of which, six miRNAs (including miR‐122, miR‐181a, miR‐146b‐5p, let‐7e, miR‐17 and miR‐143) were significantly up‐regulated from non‐neoplastic colonic mucosa to dysplasia but down‐regulated from dysplasia to tumour formation.^116^ Afterwards, more non‐coding RNAs were found to be differentially expressed and closely correlated with each step of CAC pathology (Table 1).^93^, ^101^, ^105^, ^106^, ^116^, ^117^, ^118^, ^119^, ^120^, ^121^, ^122^, ^123^, ^124^, ^125^, ^126^, ^127^ These non‐coding RNAs are either up‐regulated as oncogenes or down‐regulated as tumour suppressors by targeting the downstream genes in the categories of inflammation, cell proliferation, death, signal transduction and malignant transition. Among these markedly changed miRNAs, the vast majority are obtained and validated from human colon, colitis or cancer tissues. In 2015, Benderska et al. found that miR‐26b expression was up‐regulated with disease progression in tissues of UC and UC‐associated cancer patients. However, authors only performed comparisons between healthy people and patients with active UC blood serum. Serum miR‐26b in IBD‐associated cancer patients was not discussed.^120^ Through the miRNA profiling between UC controls and dysplastic lesions, Lewis et al. discovered 22 miRNAs that were up‐regulated in dysplasia and eight miRNAs that were maintained at relatively lower levels in UC‐dysplasia lesions. More importantly, non‐invasive biomarkers in blood between non‐cancer and cancer patients were first evaluated, and serum miR‐423‐3p and miR‐28‐5p were shown at higher levels, whereas miR‐150‐5p and miR‐32‐5p were significantly reduced in UC‐dysplasia patients.^122^

**TABLE 1:** The clinical application of non‐coding RNAs (ncRNAs) in colitis‐associated cancer (CAC) patients

Author/year	Sample source	Experimental group	Control group	ncRNAs	Up/down‐regulation	Diagnosis/prognosis	Reference
Kanaan/2012	Tissue	Dysplasia in CD patients	Non‐neoplasia in CD patients	miR‐122, miR‐181a, miR‐146b‐5p, let‐7e, miR‐17, miR‐143	Up‐regulation	–	^116^
		Cancer in CD patients	Dysplasia in CD patients	miR‐122, miR‐181a, miR‐146b‐5p, let‐7e, miR‐17, miR‐143	Down‐regulation	–	^116^
		Dysplasia in UC and CD patients Cancer in UC and CD patients	Non‐neoplasia in UC and CD patients Dysplasia in UC and CD patients	miR‐193b, miR‐373, let‐7e, miR‐15b, miR‐372	Down‐regulation	–	^116^
Olaru/2013	Tissue	Unaffected colon specimens from IBD patients and normal colonic mucosa	IBD, IBD‐dysplasias and IBD‐carcinomas	miR‐224	Up‐regulation	Diagnosis	^117^
Ludwig/2013	Tissue	Normal colonic mucosa samples Active UC	Active Crohn's colitis, UC‐low‐grade dysplasia, Crohn's colitis‐low‐grade dysplasia, UC‐high‐grade dysplasia and Crohn's colitis‐high‐grade dysplasia UC‐low‐grade dysplasia and UC‐high‐grade dysplasia	miR‐21	Up‐regulation	–	^118^
Bao/2014	Tissue	Cancer and colitis tissues	Adjacent normal colon mucosa	miR‐138, miR‐145, miR‐146a, miR‐150	Down‐regulation	–	^119^
Benderska/2015	Tissue	UC and UC‐associated colorectal carcinoma patients Active UC patients	Inactive colitis patients Healthy people	miR‐26b	Up‐regulation	–	^120^
	Serum						
Shi/2016	Tissue	UC‐associated dysplasia and UC‐associated cancer	Normal mucosa	miR‐21	Up‐regulation	–	^105^
Yang/2016	Tissue	UC patients (at diagnosis)	UC patients (at follow‐up)	miR‐21, miR‐215	Up‐regulation	–	^121^
He/2017	Tissue	IBD and CAC patients	Healthy donors	miR‐301A	Up‐regulation	–	^93^
Lewis/2017	Tissue	UC patient with dysplasia	UC patients without dysplasia	miR‐200b‐3p	Up‐regulation	–	^122^
	Serum	UC dysplasia	UC dysplasia	miR‐423‐3p, miR‐28‐5p	Up‐regulation		^122^
		UC dysplasia	UC dysplasia	miR‐32‐5p, miR‐150‐5p	Down‐regulation		^122^
Pekow/2017	Tissue	Neoplastic tissue in UC patients	Tissue from UC patients without dysplasia	miR‐31, miR‐34a, miR‐106b	Up‐regulation	–	^123^
		Neoplastic tissue in UC patients	Tissue from UC patients without dysplasia	miR‐193a‐3p	Down‐regulation	–	^123^
Pekow/2017	Tissue	UC with dysplasia	UC without dysplasia	miR‐4728‐3p	Down‐regulation	–	^124^
Ma/2018	Tissue	CAC malignant tissues and CAC‐adjacent tissues	Healthy individuals	miR‐18a	Up‐regulation	–	^106^
Feng/2018	Tissue	CAC tissues in CAC patients	Adjacent non‐cancerous tissues in CAC patients	miR‐449a	Down‐regulation	–	^125^
Song/2020	Tissue	UC with high‐ or low‐grade dysplasia	UC	miR‐31	Up‐regulation	–	^126^
Quintanilla/2022	Tissue	Ulcerative‐associated colorectal cancer and ulcerative colitis‐associated dysplasia	Ulcerative colitis‐associated normal mucosa	miR‐31, miR‐106a, miR‐135b	Up‐regulation	Diagnosis	^127^
Wan/2022	Tissue	CAC patients	UC patients and healthy controls	hsa_circ_0124022, hsa_circ_0124028, hsa_circ_0124029	Up‐regulation	Prognosis	^101^

Abbreviations: CD, Crohn's disease; IBD, inflammatory bowel diseases; UC, ulcerative colitis.

In the literature, non‐coding RNAs have also been investigated singly or in panels to evaluate their diagnostic and prognostic potentials in CAC patients. A study from Olaru et al. in 2013 reported that tissue miR‐224 expression levels increased gradually at each stage of IBD progression, and the receiver–operating characteristic curve analysis was employed to assess the potential utility of miR‐224 as a novel neoplastic disease biomarker in patients with IBD.^117^ Recently, Quintanilla et al. positively confirmed that three miRNAs’ expressions (miR‐31, miR‐106a and miR‐135b) in the colorectal tumourigenesis were associated with UC, and all these miRNAs could act as molecular markers to improve the determination of UC‐associated dysplasia.^127^ Compared to other non‐coding RNAs or mRNAs, circRNAs have covalently closed structures without 5′−3′ polarity and a polyadenylated tail, which make them relatively conserved, stable and more suitable for clinical candidate markers. Our clinical data showed that three PRKAR2A‐orignated circRNAs (hsa_circ_0124022, hsa_circ_0124028 and hsa_circ_0124029) were all significantly up‐regulated in CAC colon tissues, when compared to UC or normal colon mucosa. More interestingly, CAC patients with high PRKAR2A‐derived circRNA expressions presented a relatively shorter duration from the UC onset to carcinogenesis, and correspondingly led to an unsatisfactory clinical OS.^101^

Nowadays, there are still some limits and challenges for the potential clinical applications of non‐coding RNAs in CAC patients. First, in addition to the information from blood or colon tissues, we still need to focus on some other sources of clinical samples. For instance, stool or other bodily fluids, including urine and ascites, have been attracting attention as novel non‐invasive tools for CRC detection and monitoring.^128^, ^129^, ^130^ Colorectal mucus is an important component of the protective intestinal barrier, and the loosened colorectal mucus may lead to severe disorders, including colitis and tumour initiation. Thus, this new method of colorectal mucus sampling can also provide highly informative material and present a simple and efficient method for IBD detection.^131^, ^132^, ^133^ Unfortunately, to our knowledge, non‐invasive detection and monitoring of non‐coding RNAs in IBD and CAC patients is still a significant clinical challenge, and only changes in several non‐coding RNAs in blood have been successfully tested.^120^, ^122^ Second, compared to some conventional protein‐derived or DNA‐based medicines, non‐coding RNA‐based therapies are also attracting increasing attention for cancer treatment. Various RNA‐based or targeted medications, such as small‐molecule mimics and inhibitors of miRNAs, have been developed and approved for clinical use.^134^, ^135^ However, despite these promising progresses, no non‐coding RNA‐related therapeutics have entered and approved for clinical use of CAC treatment. Third, as discussed above, non‐coding RNAs can be classified into different classes. Unfortunately, to date, only a limited types of non‐coding RNAs, including lncRNAs, circRNAs and miRNAs, highly exist in colon tissues or serum of CAC patients and are capable to work as diagnostic or prognostic indicators, while the roles of other non‐coding RNAs, such as snoRNA, tRNA, piRNAs and pseudogenes, have not been reported. Therefore, efforts are still needed to identify novel non‐coding RNAs, especially those that are of great value for the use as clinical markers. Although there are many difficulties, we still believe that more emerging technologies and newly developed approaches will contribute to even better outcomes.

## 4 CONCLUSION

Although the exact aetiology of CAC is not completely understood, over the years, both animal models and human cohort data have implied that multiple factors, such as genetic susceptibility, barrier dysfunction, immune dysregulation and gut microbiota perturbations, contribute to the pathogenesis of CAC. To our knowledge, this is first review to summarise the aberrations and roles of non‐coding RNAs during the CAC development through various known and unknown mechanisms. Non‐coding RNAs can modulate MSI, CIN or DNA/RNA methylation to regulate oncogene or tumour suppressor expressions. Furthermore, other potential effects of non‐coding RNAs on intestinal microbiota dysbiosis, intestinal immunity, epithelial barrier and modulation of signalling pathways are also systematically discussed and confirmed. In addition, non‐coding RNAs can be determined in the colon tissue and blood and become a new source of invasive or non‐invasive tumour biomarkers for diagnosis/prognosis of CAC patients. It is also believed that, with the deepening of research, the connections between non‐coding RNAs and CAC will be increasingly studied, and more prominent biomarkers will be explored. Personally, the ideal future perspective for non‐coding RNAs as biomarkers is their utility as a minimally or non‐minimally invasive screening tools in rectal biopsy, mucus, stool or blood to identify and distinguish CAC patients.

## CONFLICT OF INTEREST STATEMENT

The authors declare they have no conflicts of interest.
